# Using a theoretical framework of Intervention Mapping to inform public health communication messages designed to increase vaccination uptake; the example of mpox in the Netherlands

**DOI:** 10.1186/s12889-023-17311-1

**Published:** 2023-11-30

**Authors:** Ymke J. Evers, Francine Schneider, Veja Widdershoven, Cornelia J. D. Goense, Charlotte M. M. Peters, Sjoerd G. van Elsen, Christian J. P. A. Hoebe, Nicole H. T. M. Dukers-Muijrers

**Affiliations:** 1 Department of Sexual Health, Infectious Diseases and Environmental Health, Living Lab Public Health, South Limburg Public Health Service, Heerlen, Limburg the Netherlands; 2 Department of Social Medicine, Care and Public Health Research Institute (CAPHRI), Maastricht University, Maastricht, the Netherlands; 3 Department of Health Promotion, Care and Public Health Research Institute (CAPHRI), Maastricht University, Maastricht, the Netherlands; 4 Soa Aids Nederland (SANL), National Knowledge Center STIs and HIV/AIDS, Amsterdam, the Netherlands; 5 Department of Medical Microbiology, Infectious Diseases and Infection Prevention, Care and Public Health Research Institute (CAPHRI), Maastricht University Medical Center+, MUMC+), Maastricht, the Netherlands

**Keywords:** Mpox, Vaccination, Prevention, Intervention Mapping, Communication

## Abstract

**Introduction:**

During an infectious disease outbreak, primary preventive pre-exposure vaccination (PPV) could substantially increase the potential for its control, if uptake is sufficiently high. An important tool to increase PPV uptake, are communication strategies, with tailored messages targeted to modify determinants for PPV uptake. Here, we take the example of the 2022 mpox multicountry outbreak, as we inform the development of communication strategies by applying a theoretical framework for selecting effective communication strategies.

**Methods:**

The theoretical framework Intervention Mapping (IM) was applied during the outbreak to inform communications [program]. Steps included: 1. Creating a logic model of the problem [not accepting PPV] by reviewing available literature, conducting an online survey among people at risk of mpox exposure, and consulting community-members, healthcare-and communication professionals; 2. Creating a matrix of change [from lower to higher PPV acceptance]; and 3. Selecting theory-based methods and practical applications for communication messages to achieve the intended behaviour change (getting vaccinated).

**Results:**

The program objective was to promote PPV uptake in people at risk of mpox exposure. Important changeable determinants identified included perceived risk and severity of mpox, importance to protect against mpox [attitude], experienced effectiveness of vaccination and side-effects [response efficacy], and social norm. Theory-based communication methods for optimizing these determinants include provision of facts [increasing knowledge], personalized risk and scenario-based risk information [addressing risk perception/severity], elaboration, arguments [stimulating a positive attitude], gain framing [increasing perceived response efficacy], guided practice [increasing skills/self-efficacy in overcoming barriers] and social norm approach [demonstrating positive norm]. Other key important factors include that communication delivery is uniform (across channels), clear, accessible, and with stigma-free messaging, and that is well-timed and repeated.

**Conclusion:**

IM provided a valuable tool in selecting communication methods to promote mpox vaccination uptake. These methods can be used to (more quickly) produce and implement a communication program in the context of possible future, vaccine-preventable, infectious disease outbreaks.

## Introduction

Vaccination is an effective strategy in controlling, vaccine preventable, infectious disease outbreaks, provided that uptake is sufficiently high [1]. Well designed and well delivered persuasive communication messages could contribute to uptake, by targeting determinants that may influence vaccination behaviour. The design of the content of these messages and strategies for their delivery are thereby vital. An example of an outbreak for which vaccination was a control strategy, is the mpox (formerly named monkeypox) multicountry outbreak that occurred in 2022 in Europe and worldwide in countries where it had not been endemic. The large number of cases, its fast spread over multiple countries and mode of transmission (sexual) was never seen before, and required immediate public health action. In this outbreak, gay, bisexual and other men who have sex with men and transgender people (GbMSM/TGP) were disproportionally affected [2, 3], and communications were targeted to this group. Various public health control measures were installed to reduce mpox spread, including early diagnosis, (self-)isolation of patients, contact tracing and post-exposure vaccination. The involvement of communities and the provision of information for behavioural risk reduction options was also vital. Several countries in Europe, Canada, and the United States, could offer primary preventive pre-exposure vaccination (PPV), by offering their scarce vaccine supplies to the group of people at highest risk for exposure. This report focuses on designing communications on the public health strategy of PPV.

The desire for fast deployment of PPV for high-risk populations in the context of the mpox outbreak has challenged healthcare professionals, policy makers, and communication officers. Under great time pressure, they needed to quickly develop and deliver effective persuasive communication messages to promote PPV uptake. It would be helpful to already have had evidence-based and theory informed communications in place, for re-use or adaption. In the context of pandemic preparedness, this report informs and facilitates the development process of effective communication strategies to promote uptake of vaccination. It does so by applying the planned and systematic approach of Intervention Mapping (IM), that allows for targeting theoretical change methods to changeable behavioural (PPV uptake) determinants, and for translating these methods into practical applications tailored to the context, needs and preferences of the focus population [4]. The IM approach guides both the development and evaluation of implementation of a communication program (or other health promotion intervention). IM consists of six iterative steps: (1) developing a logic model of the problem, (2) identifying program outcomes and objectives, (3) selecting intervention methods, (4) integrating methods and practical applications into an organized program, (5) planning for program adoption, implementing and sustainability, and (6) planning for evaluation [5]. All steps integrate behavioural theories, expert opinions, needs of the focus population, and (practical) evidence using the Core Processes [6]. Development of communication messages can be a time-consuming process, and IM has previously been proven to be an efficient tool, by outlining these Core Processes to develop messages fast and with sufficient empirical and theoretical support, and engendering community engagement [7].

In this paper, we describe the use of IM in an outbreak setting, to develop communication strategies, and apply the case example of mpox. The process and results are described regarding the design of communication strategies to promote PPV uptake among people at high risk of mpox exposure This description is intended as a model for use in future program development in infectious diseases outbreaks in focus populations, where vaccination can be a key public health control measure.

## Methods

An overview of the six IM steps can been found in Table 1. In this paper, we focus on describing and applying Steps 1–3, which are concerned with finding methods to inform the development of communication messages during the mpox outbreak.The authors of this paper did not carry out IM step 4–6 to produce a program, but we briefly described these steps to inform the production and evaluation of the program.

**Table 1:** IM step 1–3 in a nutshell, applied to an infectious disease outbreak

In this case example: the intervention (program) are communication messages; the targeted behavioural outcome is vaccination uptake
Step 1: Logic model of the problem
• Create a partnership with healthcare professionals, communication officers, policy makers and representatives of the focus population • Assess the outbreak and physical and psychosocial impact on public health and individual quality of life • Identify available preventive measures, such as vaccination • Formulate the program objective, stating what is needed to increase uptake of preventive measures • Assess behavioural and environmental causes of not using these preventive measures • Create a logic model of the problem
Step 2: Matrices of change objectives
• Formulate behavioural outcomes, stating which preventive behaviours [vaccination uptake] are needed in which population • Formulate performance objectives, stating which sub-behaviours are needed to reach the outcome behaviour (vaccination uptake) • Select important and changeable determinants of behavioural outcomes [vaccination uptake] • Combine performance objectives with determinants in change matrices, resulting in specific and measurable change objectives, stating sub-steps to reach the performance objectives
Step 3: Selection of change methods
• Selection of theory- and evidence-based methods to change the determinants of the health behaviour [vaccination uptake] and to address organizational, community and societal factors to affect the environment • Translate methods into practical applications to ensure that change methods are useable and tailored to the current outbreak context

The work of this paper, and its (intermediate) results, were at the time of the 2022 outbreak directly communicated (by N.D; Y.E) to the experts in charge of the mpox communication program nationally. These experts included the Dutch Public Health Institute for Public Health and Environment (RIVM), and the national expertise organisation STI AIDS Netherlands. Thereby, this work could directly contribute to optimisation of the design and delivery of communication messages during the outbreak.

Within each step of IM, the ‘Core Processes’ were used to identify the important literature, apply appropriate theories and collect essential additional research data [6]. In all steps, consultancy of experts and community involvement are necessary. Therefore, a partnership between researchers and experts in the field of communication, sexual health and infectious diseases, intervention development and implementation science, psychology and epidemiology has been established from the start:

- An epidemiologist in the field of sexually transmitted and other infectious diseases
- A senior researcher in the field of sexual health and prevention
- A senior researcher in the field of health promotion and intervention mapping
- A senior researcher in the field of infectious disease epidemiology and control
- A researcher in the field of vaccination hesitancy
- Infectious disease specialists (doctors and nurses)
- Communication experts from the national STI/AIDS organization and RIVM
- Community representative (from MSM community board)

### Step 1: develop a logic model of the problem

In step 1, the health problem was analysed, followed by an exploration of vaccination behaviour and related determinants. The aim was to create a Logic Model of the problem, (a) showing the health problem, (b) related behaviours of focus population (and including the environment context), and (c) reputed modifiable determinants of these behaviours. This model can be used to formulate objectives stating what is needed to change these determinants in the next step. We started by consulting sexual health nurses, infectious disease physicians, and prevention specialists, and conducted a literature review to describe the health problem related to the mpox outbreak and available measures for prevention. For the non-systematic literature review, we searched studies published in peer-reviewed journals via PubMed or retrieved from governmental databases (GOV.UK). The novelty of the health problem required a broad search to identify all relevant literature about mpox and hereby gaining insight into the experienced health complaints, affected populations and available preventive measures. Therefore, the key term used was mpox (and all related terms) and studies were included from 2022 onwards to ensure the studies were related to the recent mpox outbreak.

At the time of performing the IM steps 1–3 (during the 2022 outbreak) scarce data were available on vaccine willingness, and no data were available on vaccine acceptance for mpox and related changeable determinants. To gather the needed information, our team conducted an online survey consisting of 59 (closed or open-ended) questions, to assess vaccine acceptance. Acceptance was measured as willingness to get vaccinated when the vaccine is offered. The survey questions were informed by a community advisory board of MSM and by multidisciplinary professional experts. Respondents were GbMSM/TGP, who were recruited by convenience sampling via social media and on site, at HIV outpatients clinics, Centers for Sexual Health, and sex-on-premises venues. Recruitment was between 20 July to 5 September 2022, in the Netherlands.

The survey assessed a range of changeable determinants on the individual level. These were derived from the Protection Motivation Theory (Rogers), Health Belief Model (Luger) and Theory of Planned Behaviour (Fishbein & Ajzen) and were assessed by beliefs statements on a five-point Likert-scale.

Needs in programmatic and organisational aspects of communication and vaccination (yielding information on changeable environmental determinants) were further measured by an open-ended question and were inductively coded by two researchers. A detailed description of the survey, measures, methods and results used are reported in Dukers-Muijrers et al. 2022 [8].

The selected scope of this paper is to provide an outline of the behavioral outcome (vaccination) in GbMSM/TGP, based on changeable determinants on the individual level. Other important changeable determinants and environmental aspects (e.g. programmatic and organisation aspects of the vaccination offer) were addressed in Step 5 and the discussion section. Furthermore, we only briefly addressed the behavioural outcome in professionals (implementation of the communications) in IM Step 4–6.

### Step 2: matrices and change objectives

In step 2, change objectives were created, which are the basis for selecting effective theory-based change methods.

Firstly, behavioural outcomes were formulated, stating which health behaviours are needed from people to prevent the health problem. Secondly, performance objectives were formulated, which are sub-behaviours needed to reach the overall outcome behaviour. Important and changeable determinants of these health (sub-)behavioural outcomes were selected. Assessment of importance of determinants was based on the outcomes of our survey. Assessment of changeability was based on general insight about behavioural change. To complete this step, we created matrices that combine the performance objectives with the determinants to create change objectives.

### Step 3: change methods

In step 3, the change objectives were linked to theory- and evidence-based change methods. A change method is a technique for influencing the determinants of behaviours and environmental conditions. We selected the best fitting change methods based on those as formulated by Kok et al. (2016) [9]. Subsequently, we described practical applications to tailor these change methods to the focus population (GbMSM) and the context in which the intervention will be conducted (an outbreak situation).

## Results

### Step 1: Logic model of the problem

#### Step 1.a. Assessment of the health problem and preventive measures

##### Epidemiology

Mpox is a zoonotic disease caused by the mpox-virus [10]. The disease is endemic in some regions of Central and West Africa. Since May 2022, outbreaks of mpox have been reported in several countries in Europe and worldwide. The rapid spread, large number of cases, and transmission mode, was never seen before. The mpox outbreak has been declared as a Public Health Emergency of International Concern by the WHO Director-General on July 23 (until May 2023) [11]. Since the start of the mpox outbreak and as of 6 December 2022, 20 934 confirmed cases of mpox have been reported from 29 EU/EEA countries, of which 1247 reported cases in the Netherlands [12]. The weekly number of mpox cases reported in the EU/EEA peaked in July 2022 and a steady declining trend has been observed since.

##### Physical health symptoms

Mpox disease may begin with a combination of the following symptoms: fever, headache, chills, exhaustion, asthenia, lymph node swelling, back pain and muscle aches. However, these systemic prodrome symptoms do not always precede the onset of rash and have been absent in up to almost 50% of cases in the 2022 outbreak [13, 14]. A centrifugal maculopapular rash starts from the site of primary infection and rapidly spreads to other parts of the body. A majority of cases presented with rash in the anogenital region and inguinal lymphadenopathy [2]. Oropharyngeal involvement including oral lesions, tonsilitis and peritonsillar abscess causing pain and difficulty swallowing, and epiglottitis affecting breathing, also occurred [15]. The lesions progress, usually within 12 days, simultaneously from the stage of macules to papules, vesicles, pustules, crusts, and scabs, before falling off. Most mpox cases experience mild to moderate symptoms typically lasting two to four weeks followed by complete recovery. A minority of cases in the 2022 outbreak have been hospitalized for management of pain or complications such as secondary skin infections, abscesses, difficulty swallowing or for isolation purposes. Severe complications are reported but rare [2]. Sporadic fatal cases have also been reported [16].

##### Psychosocial impact

Previous outbreaks of infectious diseases, such as HIV, Ebola and COVID-19, have contributed to development of a variety of mental health concerns [17, 18]. The mpox outbreak was accompanied by health-stressors such as fear, panic, anxiety, anger, exhaustion, social isolation, financial loss, and importantly, also stigma [19]. The expectation and the experience of stigma was crucial. For example, this led to changing the diseases name from ‘Monkeypox’ to ‘mpox’. Also, the uncertainty surrounding a new infectious disease likely caused mental stress [19].

##### Preventive measures

Several public health control measures have been installed during the mpox outbreak. These include early diagnosis, (self-)isolation of patients, contact tracing and vaccination of contacts, and PPV using smallpox vaccines. Due to scarce vaccine supplies, PPV was offered only to a small group of individuals at high risk of exposure to mpox. To organize access, eligibility criteria for a PPV offer were formulated in several countries in Europe, Canada and the United States. In the Netherlands, GbMSM/TGP at high risk for mpox were invited for vaccination by personal email or letter, based on patient registries of the public health Centres for Sexual Health (CSH), HIV outpatient clinics, or GPs. People eligible for PPV were GbMSM/TGP participating in (or on a waiting list for) the national pre-exposure prophylaxis program for HIV (HIV-PrEP), were living with HIV and deemed at mpox risk (when they tested for hepatitis C, as a proxy for risk behaviour, or when deemed at risk by the HIV-nurse), or had according to a CSH registry in the past six months an STI diagnosis (syphilis, gonorrhea, or chlamydia), was notified for STI/HIV, or had more than three sex partners according to the national eligibility criteria in 2022. Several studies in Europe indicated a high willingness to accept mpox vaccination among GbMSM/TGP when they would be invited (70 to 85%) [8, 20, 21]. Actual uptake of PPV is yet unknown, as there are no reliable data on the number of invited people and vaccinated among invited.

The health problem was defined as the physical and psychosocial health burden of mpox among GbMSM/TGP at high risk of exposure to mpox. The related behavioural problem addressed is low acceptance and hereby suboptimal uptake of PPV among people at high risk of mpox who are actively offered PPV in the Netherlands.

##### Program objective

The objective of our program was to enhance mpox vaccination uptake among people at high risk of mpox exposure and who were eligible for PPV (according to the ruling 2022 national eligibility criteria). Still, as the here included evidence to build the program was based on the wider focus group of people at risk of mpox, including those who were not offered PPV, the information in this report is also applicable to the broader group of GbMSM/TGP at risk and a situation in which priority criteria for PPV were to be changed.

#### Step 1.b. Identification of behavioural and environmental causes of the health problem

##### Risk behaviour: not vaccinating against mpox

The risk behaviour is defined as not accepting vaccination and subsequently not getting vaccinated for mpox after a PPV offer. Since the behaviour could not be observed at the time of the current study (around the start and early PPV roll-out), the key determinant for behaviour was measured, which is acceptance, or willingness to act on the behaviour in a given situation (here: being offered PPV). Survey results demonstrated a high acceptance, i.e. 82%. Several subgroups were identified to be slightly less likely to accept vaccination, including people living in less urbanized areas, people without mpox-vaccinated social network members, and people who lacked social connection to GbMSM/TGP community.

#### Step 1.c. Changeable determinants at the individual level

Determinants related to a health problem, in this case unwillingness to vaccinate against mpox among people at risk for mpox, include determinants that can likely be influenced by targeted communication messages. The survey identified the following key determinants, i.e. perceived risk and severity, concerns about mpox, perceived importance (attitude) to protect against mpox, perceived response efficacy of vaccination (that vaccination protects against disease), trust in governmental/public health information about mpox vaccine and the perceived social norm, to be associated with vaccine acceptance [8]. All these determinants were reported with high scores by most survey-participants [8], meaning that the room for improvement of these determinants was likely limited at the time of the outbreak (when the survey was conducted). Risk perception as well as the other determinants may change with a changing epidemiological situation, which has consequences for the communication messages and its targeted determinants. For example, to promote vaccination uptake in situations where disease cases have declined, communication strategies may focus on maintenance of enduring positive attitudes towards vaccination in people with a risk for exposure.

The survey’s open questions, further identified stigma as important. For example, survey respondents suggested to use messaging that related mpox to behavioural exposure risks and networks of gbMSM/TGP people who were disproportionally affected rather than to specific population groups (e.g. framing as ‘gay disease’). The available literature shows that various types of stigma represent a major barrier to health-seeking behaviour [22]. Stigma has been associated with the actual experience of and the fear of discrimination and negative societal attitudes because of a particular condition [22]. Compared to the general population, marginalized groups, including GbMSM/TGP, are often subjected to higher levels of stigma [23]. Venereal and dermatological diseases are often stigmatized, especially those causing visible disfigurements. Various types of stigma, such as self-stigma, public stigma, and stigma from healthcare providers, contribute to health vulnerabilities, and undermine the implementation of public health interventions, such as PPV [23].

An open-ended question in the online survey revealed opportunities for improvement in the communications used at the time (of the survey during the outbreak). These included unclear or missing information about:

- the development and longer history of the vaccine that was offered.
- effectiveness and side-effects of the vaccine.
- the public health goal of the mpox vaccination campaign.
- who is invited (and who is not), and when for mpox vaccination (program planning and eligibility criteria).
- about preventive options for mpox other than vaccination.

This open-ended question revealed further issues with the communications at that time, including:

- conflicting information about mpox and vaccination operations at different information channels and from different healthcare providers in various geographical areas.

Another open-ended question revealed environmental determinants that hampered vaccination uptake, which included:

- the lack of an option to make your own appointment for vaccination (at time and location that is [more] feasible) or to self-register for vaccination (rather than to wait for a personal invitation-letter), inconvenient/longer travel distance to clinics to get vaccinated and limited available time slots.

In practice, communication officers were challenged to formulate communication messages on vaccine effectiveness, since the evidence was evolving and there was uncertainty about exact vaccine effectiveness. From the available research evidence, it is known to be important to communicate various different preventive options that people may have to minimize risk, including advantages and disadvantages of these options, while being open about what is already known and (still) uncertain (and requires further study). Further, it is known that uniformity in messages is important for building trust. However, it is a challenge to maintain uniform communication messages throughout. For example, respondents of the survey [8] indicated that general practitioners provided different information about mpox vaccination than Public Health Service STI clinics, which was found confusing.

**Fig. 1:**
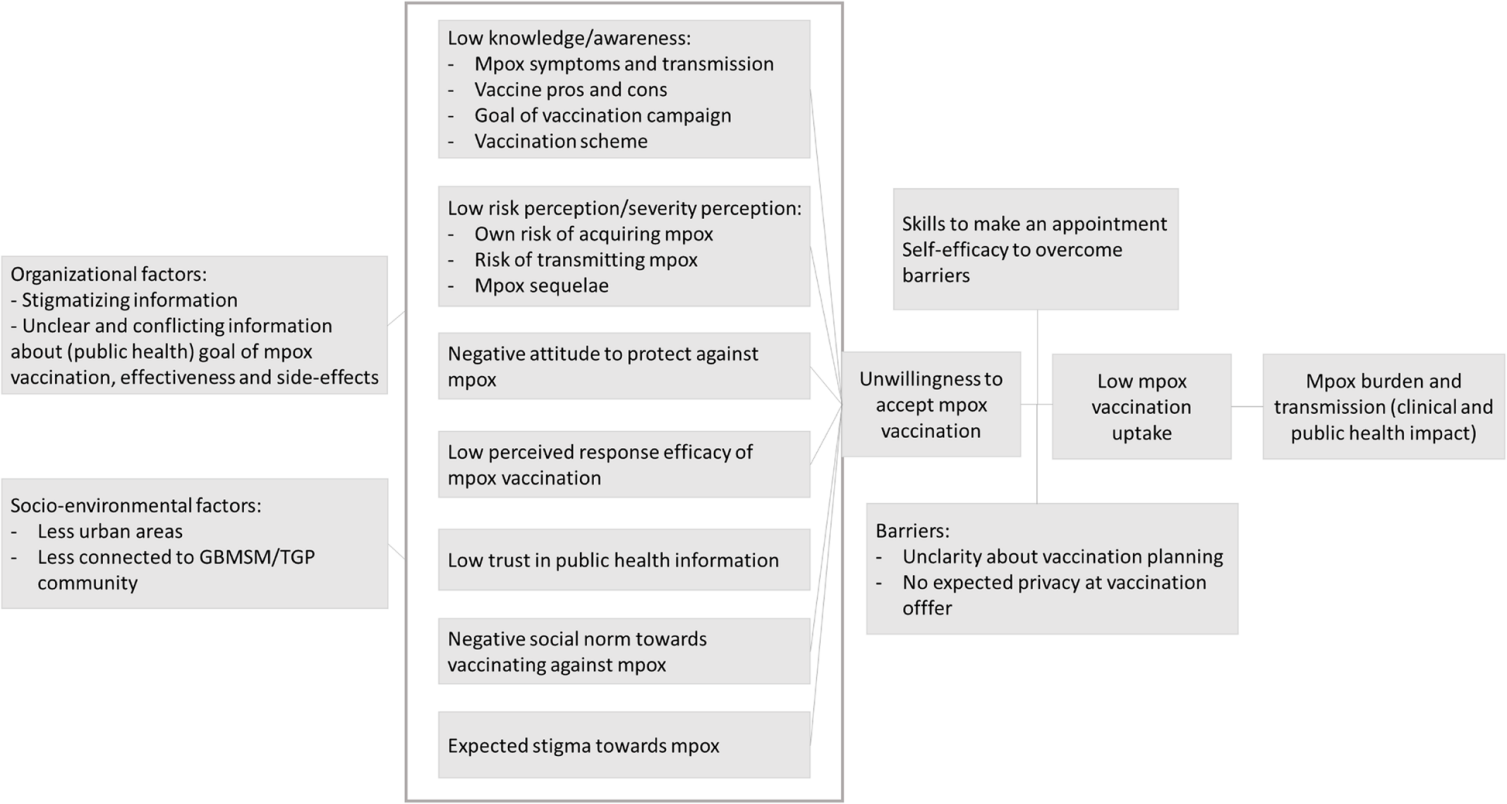
Logic model of the problem regarding mpox burden and related behaviour and determinants

Based on the Protection Motivation Theory, Health Belief Model and Theory of Planned Behaviour, the identified determinants, and the above mentioned challenges and barriers, we created a logic model of the problem as shown in Fig. 1.

### Step 2: Matrices of change objectives

#### Outcomes, performance objectives and change objectives

To promote mpox vaccination uptake among individuals at high risk of mpox exposure (eligible to receive vaccination), the following behavioural outcome was formulated: person at high risk for exposure and/or for transmitting mpox gets vaccinated against mpox. Three performance objectives (PO) were formulated: person at risk decides to get vaccinated against mpox (PO1), person at risk makes an appointment or accepts the appointment for mpox vaccination (PO2), and person at risk goes to vaccination-offer to get the vaccine (PO3). Based on the outcomes of our online questionnaire and previous studies, the most relevant and changeable determinants for each PO were selected: knowledge and awareness, risk perception (susceptibility and severity), attitude (perceived importance of vaccination), perceived response efficacy, skills and self-efficacy to overcome barriers and perceived social norms (Table 2). When targeting these determinants with communication messages, the vital aspects ‘stigma’ and ‘trust’ were integrated, by having all information being stigma-free (stigma-reducing) and having trusted sources deliver the communication-messages and in a uniform way across channels. Examples (indicated by survey respondents) include to ‘acknowledge that mpox is transmitted by specific behaviour and is not gender and sexual identify associated’ and to ‘using a credible source for the target group involving GbMSM/TGP themselves. Furthermore, we identified changeable environmental factors. These include organisational barriers, e.g. no possibility to self-register or make your own vaccination appointment at a suitable time or location, and a lack of interprofessional alignment in information-provision resulting in conflicting information and confusing messaging. These environmental factors are used to formulate the performance objectives for healthcare and communication professionals in step 5 (implementation plan), not further detailed in this paper.

**Table 2:** Change matrices for getting vaccinated (based on determinants targeted to increase willingness to get vaccinated)

PO	Awareness/knowledge	Risk and severity perception	Attitude towards protecting against mpox	Perceived response efficacy	Skills & self-efficacy to get mpox vaccintation	Perceived norm/social influence
PO1: Person who has behavioural risk for mpox exposure decides to get vaccinated	Describes what mpox is and how it is transmitted Acknowledges that mpox is transmitted by behaviour and is not associated with gender and sexual identity Describes the epidemiological situation of mpox in the Netherlands States the goals of the mpox vaccination strategy States that mpox vaccination is recommended for people who have behavioural risk for mpox Acknowledges that mpox vaccination is available only for some people because of scarcity of the supply	States negative health effects of mpox States personal susceptibility of getting mpox based on accurate behavioural risk	Acknowledges the importance of protecting oneself against mpox Acknowledges the importance of protecting others against mpox Acknowledges that it is beneficial to offer mpox vaccination to people who have behavioural risk for mpox	Lists current evidence about effectiveness and side-effects of the mpox vaccination Explains that getting mpox vaccination reduces the risk of getting (severe) mpox Explains that getting mpox vaccination reduces the risk of transmitting mpox to others Describes that getting mpox vaccination reduces the risk of having to isolate after infection Expresses the belief that there are more benefits of mpox vaccination than disadvantages	Expresses confidence in acquiring factual information on effectiveness and side-effects of mpox vaccination	Expresses the belief that other people in their social networks consider mpox vaccination as important Expresses the belief that other people in their social networks also vaccinate against mpox Expresses the belief that people in their social networks provide social support to make the decision to get mpox vaccinated Believes that healthcare professionals support the decision to get vaccinated against mpox
PO2: Person makes an appointment for vaccination	Describes where information about mpox vaccination offer can be retrieved Describes how an appointment for mpox vaccination can be made				Demonstrates the skills to make an appointment for mpox vaccination Expresses confidence in making an appointment for mpox vaccination	
PO3: Person goes to the vaccination-offer to get vaccinated	Describes where the mpox vaccination can be received Describes when the mpox vaccination can be received				Expresses confidence in going to the location to get mpox vaccinated Expresses confidence in maintenance of privacy at mpox vaccination location Expresses confidence in overcoming fear for negative reactions when going to the mpox vaccination location Expresses confidence in overcoming practical barriers (time-, location- and fear for negative reactions- related)	Expresses the belief that people in their social networks provide social support to get vaccinated against mpox

### Step 3: Change methods

#### Change methods linked to change objectives

The selected theoretical change methods, linked to the selected changeable determinants, for people at high risk of mpox are described in Tables 3 and 4. For example, consciousness raising is used as the main theoretical change method to increase people’s knowledge about mpox signs and symptoms, the epidemiological mpox situation at the time, and the public health goal of the mpox PPV campaign. Information should be understandable, clear, non-stigmatizing, uniform and timely, be provided repeatedly, and come from multiple trusted sources. Using the Elaboration Likelihood Model, central information processing is stimulated, resulting in more stable and enduring positive attitudes towards vaccination. This is done by addressing cultural similarity, reasoning and arguments using a set of meaningful premises and a conclusion of why mpox vaccination is beneficial for oneself and the community. This adds meaning to the information that is processed by providing personally relevant, surprising, repeated, and easily understandable information, using characteristics of the focus population in source, message and channel. A last example is the Social Norm Approach to stimulate a positive social norm towards vaccinating against mpox, by providing information about the proportion in the community, willing to get vaccinated, actually is vaccinated, or that supports vaccination.

**Table 3:** Change methods linked to performance objective 1

**Determinant PO1**	**Change objectives PO1**	**Method**	**Practical applications**
Awareness/knowledge	Describes what mpox is and how it is transmitted Acknowledges that mpox is transmitted by behaviour and is not gender and sexual identity associated Describes the epidemiological situation of mpox in the Netherlands States the goals of mpox vaccination strategy States that mpox vaccination is recommended for GBSM who have behavioural risk for mpox Acknowledges that mpox vaccination is available only for some people because of scarcity of the suppply	Consciousness raising (Health Belief Model)	Providing information about the causes, symptoms, current mpox situation (epidemiology), and the goal of the mpox vaccination strategy, including the rationale for triage criteria, that is understandable and factual, non-stigmatizing, complete, uniform and in time
Risk and severity perception	States negative health effects of mpox States personal susceptibility of getting mpox	Personalize risk; Scenario-based risk information (Precaution-Adoption Process Model)	Providing information about what happens when you get mpox, providing information about personal risks based on behaviour that is personally relevant and interactive
Attitude towards protecting against mpox	Acknowledges the importance of protecting oneself against mpox Acknowledges the importance of protecting others against mpox by vaccinating Acknowledges that it is beneficial to offer mpox vaccination to people who have behavioural risk for mpox	Arguments, Repeated information, Elaboration, Cultural (Communication-Persuasion Matrix; Elaboration Likelihood Model), Gain framing; Risk communication	Using a set of meaningful premises and a conclusion (based on evolving evidence) of why mpox vaccination is beneficial for oneself and the community, and add meaning to the information that is processed by providing personally relevant, surprising, repeated, and easily understandable information, using characteristics of the GbMSM/TGP (focus population) in source, message and channel Communicate the different preventive options to minimize the risk, with their advantages and disadvantages, and also be open about what is already known and what should be further studied (uncertainties), preferably in dialogue with the focus population
Perceived response efficacy & trust in information mpox vaccination	Lists the effectiveness and side-effects of the mpox vaccination Expresses the belief that there are more benefits of mpox vaccination than disadvantages Expresses the belief that getting mpox vaccination reduces the risk of getting (severe) mpox Expresses the belief that getting mpox vaccination reduces the risk of transmitting mpox to others Expresses the belief that getting mpox vaccination reduces the risk of having to isolate after infection	Gain framing, Arguments, Elaboration, Cultural similarity (Elaboration Likelihood Model)	Providing information about the gains for someone when taking the mpox vaccination, imagery about avoiding negative consequences of mpox, using a credible source for the target group (involving GbMSM/TGP themselves)
Skills & self-efficacy to get mpox vaccination	Expresses confidence in acquiring factual information on effectiveness and side-effects op mpox vaccination	Guided practice (Social Cognitive Theory)	Demonstration of someone retrieving information about mpox and talking about it
Perceived norm/social influence	Expresses the belief that other people in their social networks consider mpox vaccination as important Expresses the belief that other people in their social networks also vaccinate against mpox Expresses the belief that people in their social networks provide social support to make the decision to get mpox vaccinated	Information about others’ approval, Modelling, Mobilizing social support, Social Norm Approach (Social Comparison Theory; Theories of Social Networks and Social Support)	Providing information about % that is willing to get vaccinated and % vaccinated against mpox in the community, prompting communication about behaviour change in order to provide instrumental and emotional social support to get mpox vaccinated

**Table 4:** Change methods linked to performance objectives 2 and 3

**Determinant PO2&3**	**Change objectives PO2 & 3**	**Method**	**Practical applications**
Awareness/knowledge	Describes where information about mpox vaccination offer can be retrieved Describes how an appointment for mpox vaccination can be made Describes where the mpox vaccination can be received Describes when the mpox vaccination can be received	Consciousness raising; Advance organizers (Theories of Information Processing)	Using schematic representations with clear pictograms to provide information about how to make an appointment, vaccination locations, and the timeline of the mpox vaccination program
Skills/self-efficacy	Demonstrates the skills to make an appointment for mpox vaccination Expresses confidence in making an appointment for mpox vaccination Expresses confidence in going to the location to get mpox vaccinated Expresses confidence in maintenance of privacy at mpox vaccination location Expresses confidence in overcoming fear for negative reactions when going to the mpox vaccination location Expresses confidence in overcoming practical barriers (time- and location- and distance)	Verbal persuasion; Guided practice; Modelling (Social Cognitive Theory)	Using credible sources for the target group, GbMSM/TGP themselves, telling how they made an appointment and showing the discrete and anonymous atmosphere at the vaccination location
Social influence	Expresses the belief that people in their social networks provide social support to get vaccinated against mpox	Mobilizing social support (theories of social networks and social support)	Stimulating talking about mpox and vaccination with a few trusted people in social network by showing role models (GbMSM/TGP themselves) talking about it

### Step 4 to 6

In step 4, the communication strategy (intervention) is designed and a production plan is created. The authors of this paper not carried out this step at the time, rather during the outbreak informed the producers of the mpox vaccination campaign and information campaign about the important target determinants and which methods could be used to (i) increase the accessibility of the vaccination offer, (ii) increase uniformity, clarity and accessibility of the information, and (iii) increase vaccination uptake. The producers are policy makers, healthcare professionals, and communication officers of national infectious diseases control and STI organizations. The authors (N.D. and Y.E.) presented the findings and suggested the Intervention Mapping derived matrices for change; we did so in various interdisciplinary meetings and also by distributing a factsheet among professionals (in Dutch https://www.ggdzl.nl/professionals/publicaties/factsheets-en-rapporten/).

Communication messages need to be visible for the focus population (GbMSM/TGP), and therefore multiple and diverse types of channels should be used to disseminate information. Such channels include not only the specific channels that only target the focus population (e.g., dating-apps), but also mainstream media, general health websites, on site venues at clinics, at places where people get together, and using specific community-based channels [8]. Alongside the mode of delivery (channels), the timing of messaging and frequency of delivery is important.

In step 5, an implementation plan is developed to ensure that implementers of the intervention adopt, implement and maintain the program (i.e. the communication messages) as intended. For this step, we formulated the following behavioural outcome: professionals inform and motivate people at high risk for mpox, in the context of eligibility, to get vaccinated. Thereby, we defined the following performance objectives: the described professionals: should provide factual, understandable, non-stigmatizing information about mpox symptoms, transmission routes and current epidemiological situation (PO1); stimulate people at risk for mpox to appraise the advantages of mpox vaccination above the disadvantages (PO2); provide honest, understandable and uniform information about the goal of mpox vaccination campaign, triage criteria and underlying reasons of this triage (PO3); provide clear and uniform (non-conflicting between regions or different healthcare providers) information about making an appointment for mpox vaccination (PO4); and remove practical barriers, by creating easily accessible appointment systems, enabling vaccination at outreach locations, and ensuring privacy at clinics (PO5).

Information in the communications may change and must be updated quickly during an outbreak. Therefore, a central coordination and steering is needed from one expert organization (such as STI AIDS Netherlands) to ensure uniformity in information across channels in different regions and from different healthcare organizations. Currently, in 2023, the number of mpox cases are low. Nevertheless, the vaccination of people at high risk of mpox exposure might remain important to prevent a potential rise of cases. For this reason, The Netherlands installed a second vaccination offer in the summer of 2023, where people could self-apply for vaccination (rather than only based on personal invitation as in 2022). This adaptation was based on research findings [8].

These outcome and performance objectives are applicable to both professionals developing a mpox vaccination campaign (programmatic information) as to professionals who are involved in organisational (vaccination) aspects or are in direct contact with clients. These outcomes and performance objectives should also ensure that identified organisational and programmatic barriers (environmental aspects) will be lowered for access to information and actual vaccination.

In step 6, an evaluation plan is developed. In IM, evaluation is continuously used in all steps to optimize the intervention. For an effect evaluation for the mpox communication strategy, changes in the specific and measurable outcomes stated in Step 2 (changes in the behavioural outcome, which is vaccination uptake, the performance objectives and the selected determinants) will need to be assessed.

As the mpox communication will be implemented in real-world and having a control group for effect evaluation is not feasible (nor ethical), a pre-post implementation evaluation of optimized communication strategies would be helpful to evaluate effectiveness.

A process evaluation is recommended to understand why (or why not) the program objectives were obtained. Process measures are closely linked to the outcomes stated in Step 5 (adoption, implementation, and continuation as intended) and the performance objectives, and could include: ‘Did healthcare professionals feel sufficiently capable to inform the focus population about mpox, about the vaccination, and for making an appointment’, ‘Did the communication reach the focus population’, ‘To what level were communication messages changed in the actual roll-out at media channels’ and ‘How did the focus population perceive the information provided in the communication messages; was it understandable, uniform, stigma-free’.

## Discussion

The 2022 mpox outbreak challenged healthcare professionals, policymakers and communication officers to develop communication messages to promote uptake of vaccination under high time pressure. Here, we demonstrated that a theoretical framework, i.e., intervention mapping, can be used to guide the process of selecting behaviour change methods that are important in increasing vaccination uptake among people at risk of mpox. This guide of systematic development thereby informs epidemic preparedness and response, for focus populations such as GbMSM/TGP. Persuasive communication strategies about mpox vaccination have been implemented in most countries that offered PPV. The here suggested change methods and practical applications to implement these methods can be applied to fine-tune the strategy for future communications (both in online campaigning and communication between professionals and clients) on mpox or other vaccine-preventable infectious diseases in a similar context. Using adaptation, and engaging the community is vital to ensure that a communication strategy is tailored to a current context and focus population.

IM helped to select persuasive communication strategies that are targeted to the important determinants of mpox vaccination uptake in a systematic way. This process was based on theory- and evidence ensuring that all relevant behavioural determinants are addressed. Individual level determinants for mpox vaccination included awareness, risk and severity perception, attitude towards protecting against mpox, perceived response efficacy, skills/self-efficacy to make a vaccination appointment, and the perceived norm. These determinants are largely in line with previous research into vaccination willingness and hesitancy [24]. The combination of expert opinions, questionnaire data, and behaviour explanation and change theories clarified which change methods and practical applications should be used to develop communication messages.

Several recommendations on methods and applications arised from this study.

I. Information about the outbreak, transmission, symptoms and the goal of the vaccination strategy should be provided in a way that is understandable, factual, non-stigmatizing, timely, and uniform across geographical regions and types of healthcare professionals. To maintain well-designed messaging is important, especially in the current infodemic era, where biased messages can spread easily and quickly. Culturally appropriate messaging will equip people and communities to protect themselves and others. The partnership with affected communities is therefore key for most effective formulation and delivery of communications, and also essential to maintaining the public’s trust [24, 25].
II. Communication officers indicated difficulty in communicating uncertainties about vaccine effectiveness. In risk communication, transparency about evolving evidence and being open about different preventive options with their advantages, disadvantages and uncertainties is important to build and maintain trust in public health policy [24].
III. People at risk should be facilitated to appraise personal risks. This can be achieved by personalized and scenario-based risk information. After appraising the risks and severity of the infectious disease, it is crucial that people have access to preventive actions decreasing these risks. Therefore, meaningful premises and a conclusion on why vaccination can be beneficial for oneself and the community should be given in a way that it is personally relevant, surprising and repeated (Elaboration Likelihood Model) [26]. This also entails the provision of information about other preventive actions, especially to people who are currently not eligible to receive vaccination due to scarce vaccine supply and related strict triage criteria. Gain framing the advantages for someone when taking the vaccination (or other preventive actions) by imagery of avoiding negative consequences of the infectious disease is an effective strategy in increasing perceived response efficacy. Gain-framed messages appeal to be more effective when targeting behaviours that prevent onset of a disease than loss-framed messages [27]. Moreover, previous research shows that tailoring communication to specific concerns and doubts of people who are hesitant about vaccination are crucial in discussions between healthcare providers and patients to increase vaccination willingness [28]. A positive social norm towards vaccination could be stimulated by providing information on the proportion of GbMSM/TGP willing to vaccinate, already vaccinated, or supporting vaccination [29].
IV. For all these methods, it is important that characteristics of the focus population are used in source, message and channel so that the information is visible and easily accessible for people at high risk of exposure. As our previous study has shown that people living less urban areas and/or feeling less connected to GbMSM/TGP communities were slightly less willing to vaccinate against mpox [8], multiple communication channels should be used to make information available, including mainstream media, general health websites, at clinics, at venues (where people get together), in addition to using specific community-based channels (e.g. gay dating apps).
V. We identified that communication strategies should include clear, non-stigmatizing, and transparent information about the vaccination scheme (who will be vaccinated and when), possibility for self-registration and make your own appointment for vaccination at a suited time and place (increasing autonomy and decreasing timing barriers), vaccination locations in and outside of the clinic (decreasing distance), and ensuring privacy at the clinic [8]. These environmental determinants were (in addition to individual level determinants) vital for vaccination uptake, as revealed by creation of the logic model of the problem and consultancy with stakeholders and the focus population. Communication strategies and vaccination organisation should focus to reduce both the expected and experienced organizational barriers to get vaccinated, to make vaccination more accessible for people who have a high willingness (intention-behaviour gap) [4]. This requires an implementation plan stating what is needed from organisations, and specifically healthcare professionals and communication officers, to effectively reduce organizational barriers and implement the communication strategies that address organisational aspects.
VI. Fast changing and growing evidence and policies regarding mpox control strategies, call for transparency and central steering of information and communication to avoid conflicting information provided from different channels, regions, and healthcare providers [8].
VII. During the outbreak, we identified high scores on the individual level determinants, leaving little room for improvement. Therefore, at that time, communication strategies would also benefit from installing frequent exposure to increase chances of ensured positive attitudes towards vaccination (later on). As our survey also showed high vaccination willingness among PPV non-eligible persons, the suggested change methods will also be relevant for a broader group in case vaccination would be more broadly available [8].
VIII. A next step would be evaluation of the effectiveness of the communications (whether these effectively changed the determinants and eventually uptake of mpox vaccination in people at high risk), and the evaluation, monitoring and optimization of the implementation of communication strategies.

### Strengths and limitations

This study provided an example of the application of the IM approach to the development of theory- and evidence-informed communication messages to increase vaccination uptake for an infectious disease, i.e. mpox. This example allows replication or further build on by future vaccination campaign developers in an infectious disease outbreak. By using the IM protocol, we selected change methods and parameters for use, which are based on theory, evidence (a survey among GbMSM/TGP), and consultancy of experts and community-members. The process described here from the systematic identification of determinants related to the health problem towards the selection of change methods to increase vaccination uptake can be used as a guide of how a intervention (here: communications) can be quickly developed during an infectious disease outbreak. The literature search has not been done systematically and we have conducted and updated the literature search at the same time as performing the recruitment and analyses if the online survey, to decrease time-lags between IM Steps [30]. Healthcare professionals and communication specialists were consulted during several stages of the process, but the collection of information was mainly unstructured and informal. Therefore, it is possible that not all perspectives were taken into account. The needs of GbMSM/TGP themselves were extensively assessed in the online questionnaire. However, in the future steps, engaging people more in the production and testing of communication materials ensures that materials will be even more tailored. Consulting existing community boards or panels, would be one efficient way to co-create communications with the focus population.

## Conclusion

Intervention Mapping provided a valuable tool in selecting effective methods to promote mpox vaccination uptake in people at risk for mpox. We identified several concrete communication methods and applications, which can be used by policy makers, communication officers and healthcare professionals in producing and optimizing a campaign to promote vaccination in the context of mpox. The description of the systematic process of developing communication strategies to increase vaccination uptake could also be useful during future infectious disease outbreaks for which vaccination is an effective control measure.

## Data Availability

All data generated and/or analysed during the current study are included in this published article.
